# Supplementary material for: Comparison of the recovery quality between remimazolam and propofol after general anesthesia: systematic review and a meta-analysis of randomized controlled trials
Source: PeerJ. 2024 Aug 26;12:e17930. doi: 10.7717/peerj.17930 (PMC11361258; doi:10.7717/peerj.17930)
Supplement: Supplemental Information 1 [file peerj-12-17930-s001.docx]

**Supplementary Table S1** The complete search strategy of PubMed

| Number | Search terms |
| --- | --- |
| 1 | Remimazolam* |
| 2 | Propofol [Mesh] |
| 3 | Propofol* |
| 4 | 2 OR 3 |
| 5 | Randomized controlled trial |
| 6 | Randomized |
| 7 | Randomly |
| 8 | random |
| 9 | 5 OR 6 OR 7 OR 8 |
| 10 | quality of recovery |
| 11 | recovery quality |
| 12 | QoR |
| 13 | 10 OR 11 OR 12 |
| 14 | 1 AND 4 AND 9 AND13 |
