# Supplementary figures and images for: Comparison of the recovery quality between remimazolam and propofol after general anesthesia: systematic review and a meta-analysis of randomized controlled trials
Source: PeerJ. 2024 Aug 26;12:e17930. doi: 10.7717/peerj.17930 (PMC11361258; doi:10.7717/peerj.17930)

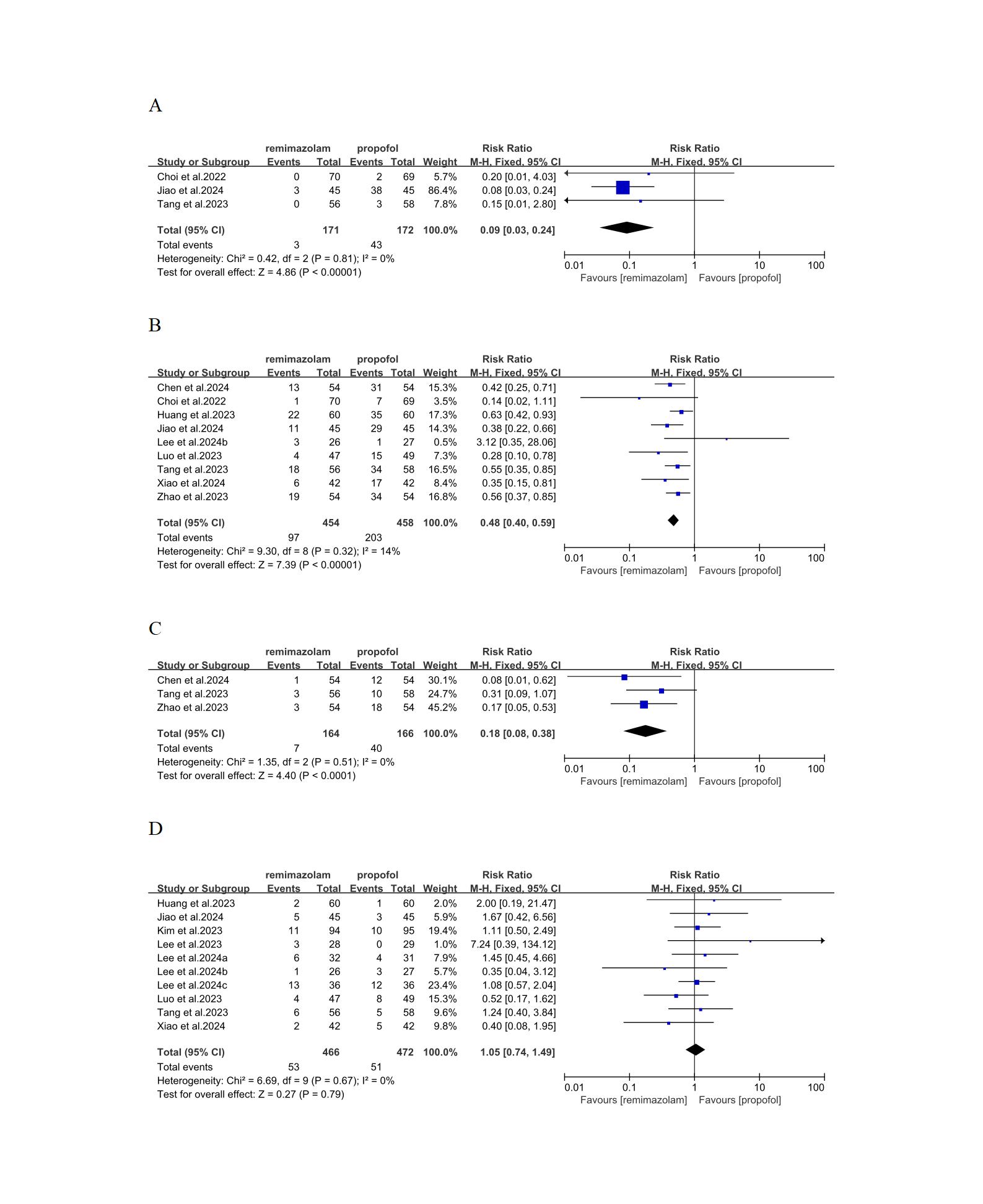

Supplement: Supplemental Information 2 — M-H, Mantel-Haenszel; CI, confidence interval. [file peerj-12-17930-s002.jpg]
